# DoE- and PBBM-Driven Formulation Development of an Extended-Release Donepezil Tablet

**DOI:** 10.3390/ph18121894

**Published:** 2025-12-16

**Authors:** Frederico Severino Martins, Leonardo Luiz Borges, Sivacharan Kollipara, Praveen Sivadasu, Renê Oliveira do Couto

**Affiliations:** 1 Institute of Technology and Research (ITP), Av. Murilo Dantas, 300, Aracaju 49010-390, Sergipe, Brazil; 2 School of Medical and Life Sciences, Pontifical Catholic University of Goiás, Goiânia 74605-010, Goias, Brazil; 3 Theoretical and Structural Chemistry Group of Anápolis, State University of Goiás, Anápolis 75132-903, Goias, Brazil; 4 Department of Pharmacy, Koneru Lakshmaiah Education Foundation, Green Fields, Vaddeswaram 522302, Andhra Pradesh, India; 5 Midwest Campus, Universidade Federal de São João del-Rei (UFSJ), Divinopolis 35501-296, Minas Gerais, Brazil

**Keywords:** design of experiments, physiologically based biopharmaceutics modeling, extended-release formulations, Box–Behnken design, virtual bioequivalence

## Abstract

**Background/Objectives**: This study explores the integration of Design of Experiments (DoE) with Physiologically Based Biopharmaceutics Modeling (PBBM) to streamline the development of extended-release (XR) formulations. Using donepezil (DPZ) as a model drug, we developed an optimized XR formulation exhibiting a dissolution profile comparable to the reference product, Aricept^®^ (Eisai GmbH, Frankfurt, Germany). **Methods**: A Box–Behnken experimental design was applied to systematically evaluate how formulation variables—HPMC 100, HPMC 4000, and NaCMC—affect drug release kinetics, tablet hydration, and erosion. This strategy enabled the identification of optimal excipient concentrations with minimal experimental effort. **Results**: The *in vitro* dissolution data were then integrated into a PBBM framework to simulate drug release and pharmacokinetics, enabling virtual bioequivalence (VBE) assessments. The combined approach provided robust predictive insights into formulation performance, substantially reducing reliance on resource-intensive *in vivo* studies. Beyond its successful application with DPZ, this integrated methodology offers a scalable and generalizable strategy for efficiently developing bioequivalent XR formulations for various clinically relevant drugs. **Conclusions**: Our findings highlight the importance of leveraging advanced statistical methods and in silico modeling to overcome contemporary pharmaceutical development challenges, paving the way for innovative, cost-effective solutions that significantly accelerate time-to-market.

## 1. Introduction

Donepezil (DPZ) is a drug used to treat Alzheimer’s disease. It works by blocking the enzyme that breaks down acetylcholine—a brain chemical important for memory and thinking—which helps improve cognitive function. Clinical studies of DPZ have been conducted to assess the efficacy of alternative formulations and routes of administration of DPZ [1,2,3,4].

The currently available reference formulations include Aricept^®^ 5 and 10 mg immediate-release (IR) tablets, approved by the USFDA in 1996, and a 23 mg extended-release (XR) tablet approved in 2010. The XR formulation is designed to maintain therapeutic drug levels over a longer period, exhibiting a slower time to reach peak concentration (3 to 8 h) compared to the IR tablets. This pharmacokinetic profile supports less frequent dosing while providing similar overall exposure [2,5,6]

A common strategy for achieving extended drug release is through hydrophilic matrix tablets. These tablets incorporate water-swellable, non-cross-linked polymers such as hydroxypropyl methylcellulose (HPMC). Upon oral administration, HPMC rapidly hydrates and forms a gel layer around the tablet core, regulating the drug release rate as it moves through the gastrointestinal tract. Hydration creates a gradient in polymer concentration that defines three distinct regions: an outer gel layer, a swollen glassy layer, and an inner dry core. This structure leads to the formation of an erosion–diffusion front and a swelling front, which together influence the drug dissolution process [7,8,9]. Due to the complex interplay of these mechanisms, developing a generic XR formulation that is bioequivalent to the reference product can be challenging and often requires multiple experimental iterations [7,10,11,12,13,14,15,16].

To streamline this process, Design of Experiments (DoE) is employed as a statistical tool to identify and optimize the critical factors affecting *in vitro* dissolution and overall drug release. DoE helps in understanding the relationship between formulation variables and their impact on the critical quality attributes, enabling a more focused approach in achieving a bioequivalent formulation [12,17,18,19].

Furthermore, combining DoE with Physiologically Based Biopharmaceutics Modeling (PBBM) allows for the early prediction of *in vivo* pharmacokinetics based on formulation changes. PBBM is increasingly used to assess bioequivalence between generic and innovator formulations, support biowaiver applications, and minimize the need for extensive food effect or fed bioequivalence studies. By integrating *in vitro* data from DoE into PBBM, researchers can perform in silico evaluations to predict the clinical performance of the XR tablet [2,18,19,20,21,22,23,24]. When PBBM is coupled with DoE, it becomes a powerful tool to design formulations that can give the desired *in vivo* performance.

This work aimed to develop a new extended-release donepezil formulation that is bioequivalent to the reference product. The approach involved using DoE for systematic formulation optimization and identification of the critical variables that govern *in vivo* performance. A validated PBBM, coupled with virtual bioequivalence testing, was then applied to assess the *in silico* behavior of the optimized formulation. This integrated strategy has the potential to reduce the reliance on costly and time-consuming human studies, thereby accelerating the development process and facilitating a faster market launch.

## 2. Results

### 2.1. Design of Experiment (DoE) Based on Box–Behnken Evaluation

A three-factor, three-level Box–Behnken design (BBD) was employed to evaluate the impact of excipients on DPZ release. The independent factors, chosen as drug release rate modifiers, were x1 = HPMC 100 cps, x2 = HPMC 4000 cps, and x3 = NaCMC, each tested at three levels. ANOVA and correlation analyses were carried out to precisely determine how these factors interact with key quality indicators. Table 1 provides a summary of the main effects along with their significance values (expressed as percentages). It also includes comments on highly significant interactions and uses the symbols (+) and (−) to denote the direction of each effect.

Response surface analysis enabled the fitting of polynomial equations to the dependent variables as functions of the significant factors, thus supporting the prediction of quality indicators. Below 5 h, factors x_1_, x_2_, and x_3_ were significant; the x_1_·x_2_ interaction was significant at 13 h; and the quadratic terms x_1_^2^ and x_3_^2^ were significant at 1 and 13 h. Additionally, x_2_^2^ was significant below 5 h.

**Figure 1:**
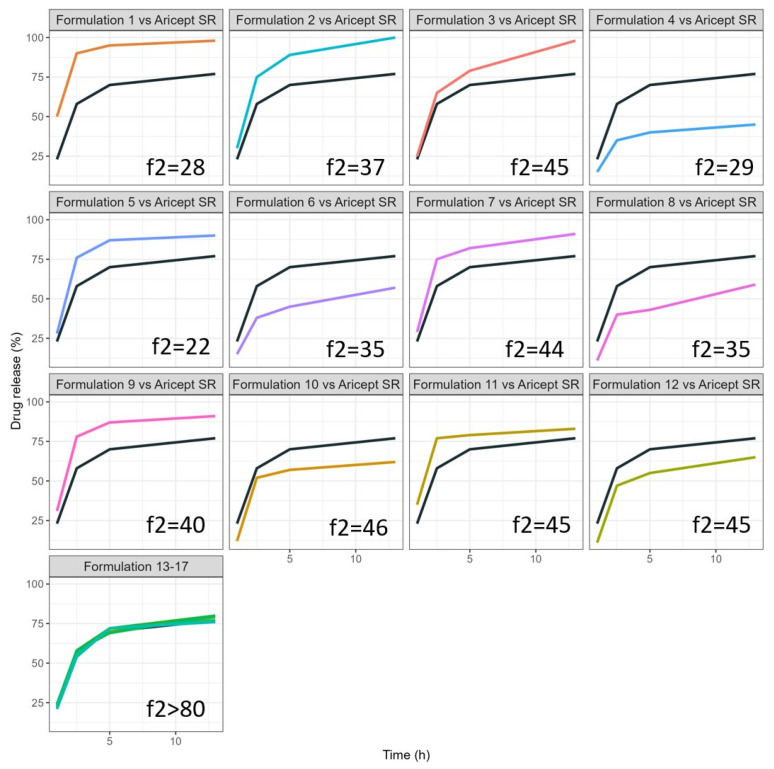
Dissolution profile of donepezil for several prototype formulations (colorful lines studied compared with Aricept^®^ formulation (black line). Note: Formulations 13, 14, 15, 16, and 17 are the replicates of the central point and overlap the Aricept dissolution curve.

Figure 1 presents the dissolution profiles for all 17 formulations. The in vitro release data were modeled using a double Weibull function, which provided good fits with determination coefficients (r^2^) of >0.97. Notably, the formulation at the central point achieved an *f*2 (pH6.8 and 100 rpm) value exceeding 50, identifying it as the prime candidate for VBE evaluation. In addition, Formulations 9, 10, 11, and 12 exhibited *f*2 values > 40 that warrant further investigation of their bioequivalence potential.

**Figure 2:**
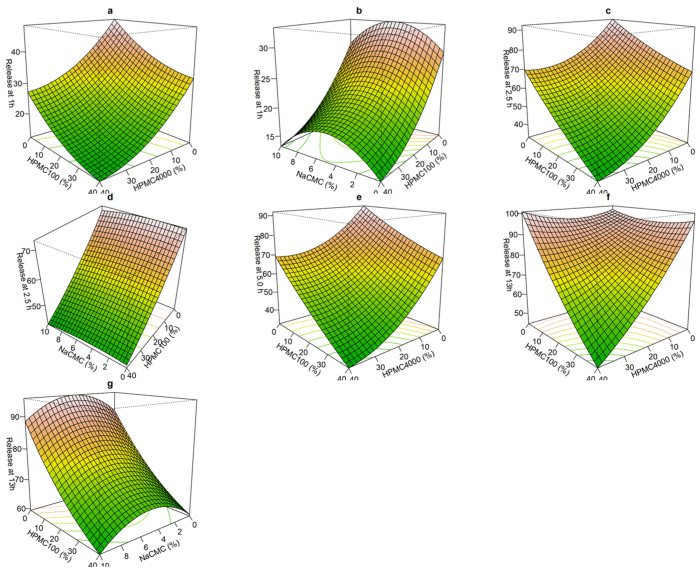
Response surface plot showing the Influence of HPMC and NaCMC on donepezil release at several times (**a**–**g**).

Figure 2 illustrates the 3D response surface analysis. The initial release rate over the first 5 h was influenced by the proportion of the release polymer, HPMC. An increase in HPMC results in a slower release rate. NaCMC exhibits a nonlinear effect on drug release at 1 h but shows no effect from 2.5 h onward. Using the Box–Behnken Design (BBD), 17 formulations were evaluated to identify the optimal release rate that aligns with Aricept^®^ (1 h = 23%, 2.5 h = 58%, and 13 h = 77%). The optimized formulation (F_optz_) is represented by the Formulations 13–17 (replicates of the central point), which contained 20% HPMC 100, 20% HPMC 4000, and 5% NaCMC, resulting in actual release rates of 1 h = 22%, 2.5 h = 55%, and 13 h = 76%.

### 2.2. Determination of Gel Strength, Hydration, and Erosion

**Figure 3:**
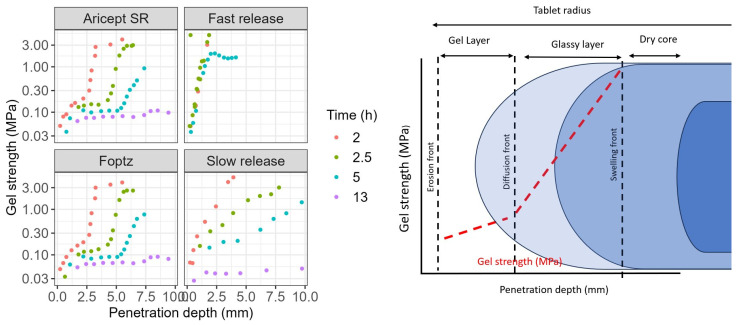
Gel strength profiles of investigated products by texture analyzer in different swelling times in the development of donepezil extended-release tablets.

The F_optz_ exhibited a hydration and gel strength profile similar to that of the reference product, with a clear delineation of the gel layer formation over time (Figure 3). In contrast, the fast-release formulation, which contained a lower amount of polymer, showed rapid hydration during the initial hours. Its gel viscosity increased within the first 2 h but decreased after 5 h, resulting in a mass loss.

**Figure 4:**
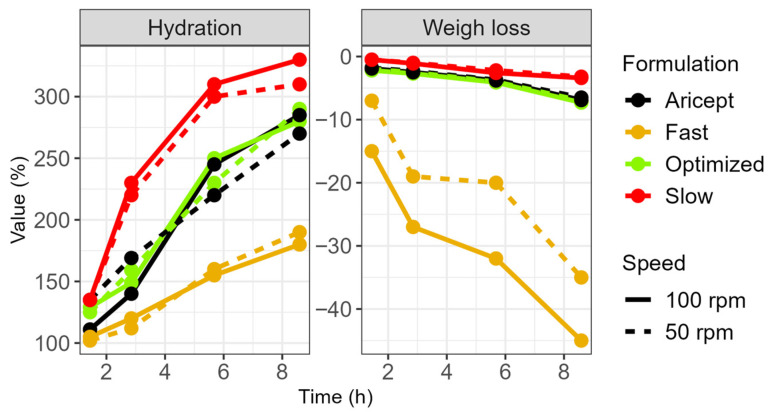
Hydration and weight loss of donepezil extended-release tablets during dissolution at different agitation speeds.

Furthermore, the fast-release tablet was more sensitive to stirring speed. At higher stirring speeds, its gel layer became thinner, thereby allowing the softened core to exert a greater influence on the erosion rate (Figure 4). Water uptake by the tablets was directly related to their HPMC content: the slow-release formulation gained 300% in weight, both the F_optz_ and the reference formulation gained 250%, and the fast-release formulation gained 175%. Additionally, a formulation containing the highest polymer content exhibited a unique relationship between gel strength and hydration time, although its profile differed from that of the commercial product.

### 2.3. PBB Model Development

**Figure 5:**
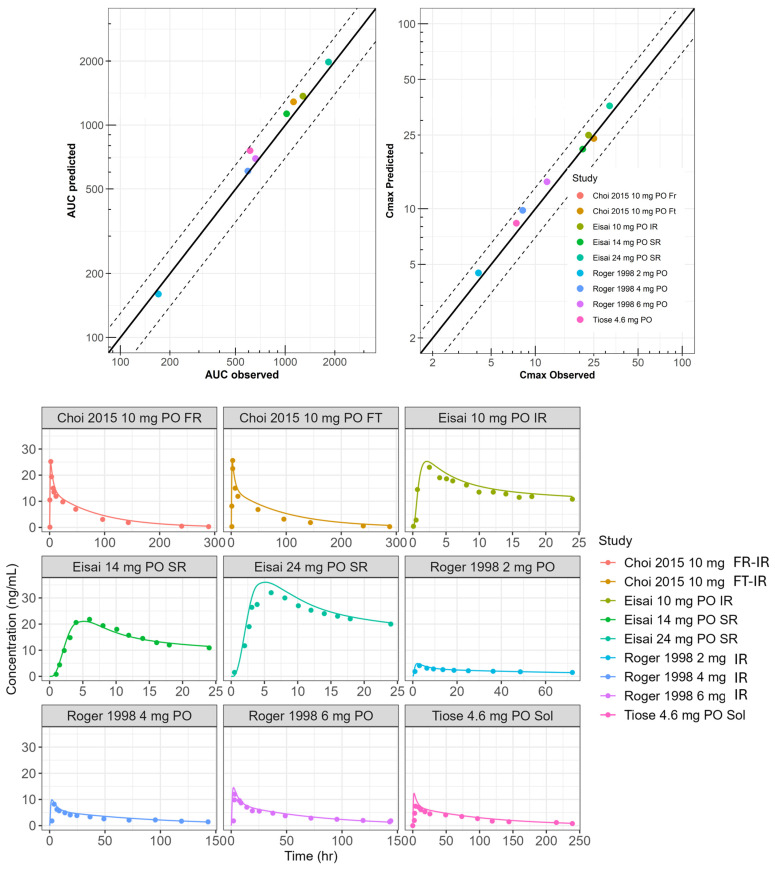
Model performance comparing observed versus predicted AUC and C_max_, along with simulated versus reported pharmacokinetic profiles from multiple sources and dose levels during the development of donepezil extended-release tablets. SR = sustained-release formulation; IR = immediate-release oral solid formulation; Sol = oral solution formulation; FR = reference formulation (IR); FT = test formulation (IR). The 4.6 mg dataset from Tiseo et al. (1998) [5] was used during model development, in which SF was adjusted to recover observed clearance, while all other datasets were used solely for model validation.

The performance of the Physiologically Based Biopharmaceutics Model (PBBM) was validated using nine published datasets, with the Average Fold Error (AFE) of predicted C_max_ and AUC_0–t_ values falling within 0.8 to 1.25 times the corresponding observed means (Figure 5).

Supplementary Material Figure S1 illustrates the simulated *in vivo* dissolution and absorption profiles for both the XR and IR formulations. The XR formulation demonstrated gradual dissolution, achieving near-complete release after 10 h, which corresponded with a delayed and sustained fraction absorbed (fa). In contrast, the IR formulation exhibited rapid dissolution and faster absorption, with the dissolution process plateauing within the first 15 min.

Further analysis of the fa across various gastrointestinal segments revealed distinct absorption patterns for each formulation Figure S1. The IR formulation achieved higher absorption in the proximal regions of the intestine, particularly in the duodenum (37%) and jejunum 1 (40.8%). Conversely, the XR formulation showed a more distributed absorption profile, with notable uptake in distal regions such as the ileum, caecum (24%), and ascending colon (6%). While the IR formulation achieved complete absorption (100%), the XR formulation reached 83%, reflecting its controlled-release characteristics and extended absorption window.

Additionally, sensitivity analysis (Figure S2 in Supplementary Materials) identified that the parameters most influencing DPZ exposure were those associated with formulation performance, intestinal permeability, gastric emptying time, and the maximum reaction rates of CYP3A4 and CYP2D6.

### 2.4. Virtual Bioequivalence

The model was systematically applied to assess the likelihood of F_optz_ being bioequivalent. The initial step involved constructing virtual populations that mirrored the between-subject (BSV) and within-subject variability (WSV) reported by Yewon Choi et al. [25], Rojanasthien et al. [26], and Manamuti et al. [27]. A WSV between 5 and 16% was observed for both AUC and Cmax. In the absence of prior knowledge on the physiological parameters contributing to WSV, the PSA results guided the virtual population development. Lastly, the sensitive parameters were tuned to replicate the BSV and WSV observed in vivo (Table 2). We compared F_optz_ and F_reference_ by incorporating their direct dissolution profiles into the simulation. At this stage, we assumed in vitro dissolution variability for the Weibull scale (Weibscale: 5.0%), shape (Weibshape: 5.0%), and Fmax (3.2%). Since we did not have a pilot or pivotal study to confirm whether the variability attributed to the formulation was accurate, we adopted a stepwise approach to determine the sample size for a future bioequivalence study. Initially, a virtual population (*n* = 18) was created using the input variabilities and multiple trials (*n* = 15), then this population showed an inter-subject variability (ISV) of 19% to Cmax and 13% to AUC.

**Figure 6:**
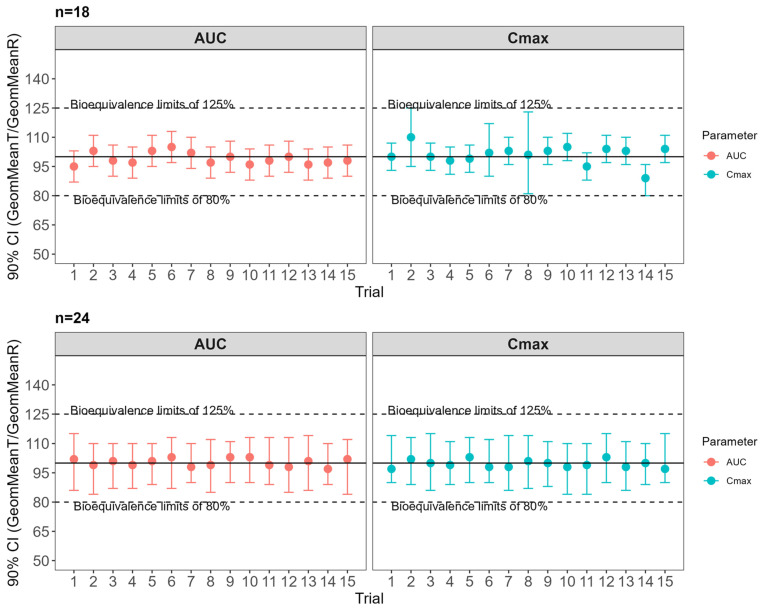
Virtual bioequivalence between the optimized formulation for extended release of donepezil and the market product Aricept^®^.

Based on the virtual WSV, a VBE study with 18 subjects would be required to achieve at least 80% power and fit within the bioequivalence (BE) intervals of 80–125%. However, the new randomized population generated by the software 3 trials did not pass the BE criteria due to the lack of power <80% (Figure 5 and Figure S3). By increasing the population size to *n* = 24, all 15 trials passed the BE criteria (Figure 6). Formulations F7, F9, F10, F11, and F12 had a lower likelihood of being bioequivalent when compared with the RLD formulation, and more than 70% of virtual trials were out of BE limits (Figure S4).

## 3. Discussion

The present study shows the benefits of combining Design of Experiments (DoE) with Physiologically Based Biopharmaceutics Modeling (PBBM) to speed up the development of an extended-release donepezil formulation. By integrating these laboratory experiments and computer simulations, we effectively tackled common challenges associated with hydrophilic matrix tablets. This strategy significantly reduced the amount of trial-and-error experimentation and minimized the need for costly bioequivalence studies in humans.

Hydrophilic matrix tablets using HPMC are well-known for controlling drug release through hydration, swelling, and erosion. Like previous studies, we found that the combination of polymer viscosity, polymer amount, and additional ingredients significantly influences drug release. Higher polymer content created a strong gel layer, slowing down the initial drug release, while lower polymer amounts or lower-viscosity polymers led to faster hydration, erosion, and quicker drug release [20,23,28].

Our results also showed that NaCMC had a dual effect on drug release, initially influencing release in a nonlinear way and later becoming linear. This is likely due to rapid initial swelling, followed by slower, erosion-based release. Other studies have similarly pointed out the importance of polymer interactions in controlling gel strength and erosion.

Using the BBD allowed us to clearly understand how different grades of HPMC and NaCMC interact, helping us quickly identify an optimal formulation matching the reference release profile. This aligns with the previous literature recommending DoE methods to handle multiple formulation factors. We developed robust statistical models highlighting important main effects, interactions, and quadratic relationships. Consistent with earlier findings, linear interactions mattered most early on, whereas quadratic effects became significant over extended dissolution periods [19,22,28].

Evaluating gel strength, hydration, and erosion provided valuable insights, consistent with previous studies [29,30,31]. Higher amounts of HPMC created stable gel layers crucial for predictable and extended drug release. The optimized formulation closely resembled the gel strength and swelling properties of the reference product (Aricept^®^), reducing the risk of dose dumping and enhancing robustness across different physiological conditions. This supports regulatory expectations for extended-release formulations.

We validated the PBBM approach using nine clinical datasets covering various doses and formulations of donepezil. The model demonstrated strong predictive accuracy, with an Average Fold Error between 0.8 and 1.25 for critical parameters like Cmax and AUC_0–t.

The sensitivity analysis identified dissolution rate, CYP2D6/CYP3A4 metabolism, and gastric emptying as critical factors impacting *in vivo* performance. These findings align with earlier reports highlighting variability due to CYP2D6 genetics and gastrointestinal transit times, reinforcing the importance of incorporating such variability into virtual bioequivalence simulations [32,33,34,35].

Virtual bioequivalence assessments showed that the optimized extended-release formulation has a strong likelihood of being bioequivalent to the reference, even considering the typical 10–16% intra-subject variability reported for donepezil. Regulatory bodies like the FDA and EMA increasingly acknowledge modeling approaches such as PBPK/PBBM in drug development guidelines [31,36,37,38,39]. Our study successfully illustrates the value of using these approaches, supporting their broader adoption in generic drug development.

Nevertheless, combining DoE with PBBM for extended-release formulations is challenging. The complexity and inherent variability of both laboratory and physiological systems make accurate modeling difficult. High-quality data on physicochemical properties, dissolution, and physiological details are not always consistently available. Additionally, PBBM predictions are sensitive to many factors, such as gastrointestinal transit, enzyme kinetics, and variability between individuals, complicating accurate modeling of human physiology. The nonlinear nature of polymer–drug interactions and the delicate balance between swelling, erosion, and gel formation further add to these complexities. Addressing these challenges requires careful model validation, precise handling of variability, and close collaboration between experimental and computational teams to ensure predictions translate reliably into clinical success and satisfy regulatory requirements.

## 4. Materials and Methods

### 4.1. Quantitative Analysis of Donepezil

The high-performance liquid chromatography (HPLC) analysis of DPZ was performed by a Shimadzu HPLC system 10 series coupled to photodiode array detector. Donepezil in the dissolution medium samples was separated on an HPLC column LiChroCART 10 × 2 mm, LiChrospher^®^ (uHPCLS, São Paulo, Brazil) 100 RP-18 5 µm. An isocratic solvent system consisting of acetonitrile, methanol, and 10 mM ammonium phosphate buffer (pH 6.8) (30:20:50% *v*/*v*/*v*) was used as the mobile phase at a flow rate of 0.2 mL/min. The sample injection volume was 15 µL, and donepezil was detected at 270 nm.

### 4.2. Design of Experiment (DoE) Based on Box–Behnken Evaluation

The impact of excipients was studied with a 3-factor, 3-level Box–Behnken design (BBD) using R studio 4.1^®^ (RStudio Team 2020-RStudio: Integrated Development for R. RStudio, PBC, Boston, MA, USA, www.rstudio.com (accessed on 10 August 2024) and the package “rsm” (cran.r-project.org/web/packages/rsm/ (accessed on 10 August 2024)). The independent variables (factors) selected as drug release rate modifiers were HPMC 100 cps (X1), HPMC 4000 cps (X2), and NaCMC (X3) in 3 levels—low (−1), medium (0), and high levels (+1)—and lactose was used as filler to q.s. 750 mg (Table S1 in the Supplementary Materials). Seventeen formulations were prepared, and 5 central points were used to determine the experimental error and the precision of the design. The dependent variables were released at 1 h (Y1), 2.5 h (Y2), 5.0 h (Y3), and 13 h (Y4). All XR tablets have fixed amounts of donepezil hydrochloride (23 mg as donepezil HCL) and magnesium stearate (0.99%).

Only variables with a significance level greater than or equal to 5% (*p* = 0.05) were taken into consideration. A quadratic polynomial equation generated by Equation (1) served as the response function that was used:

(1) $$Y\;=\;\beta_{0}+\sum_{i\;=\;l}^{K}\beta_{i}X_{i}+\sum_{i\;=\;l}^{K}\beta_{ii}X_{ii}^{2}+\sum_{i\;=\;l}^{k\;=\;1}\sum_{j\;=\;2}^{k}\beta_{ij}\;X_{i}X_{J}$$

where *Y* is the predicted response (dependent variable); *β*_0_ is the model constant; *X_i_* and *X_j_* are independent variables; *β_i_* and *β_j_* are the linear coefficients; and *β_ii_* and *β_ij_* are the quadratic coefficients.

### 4.3. Preparation of Donepezil XR Tablets

The donepezil XR tablets were prepared following a wet granulation method, and the API (active pharmaceutical ingredient) was mixed with lactose (filler), HPMC 100 cps, and 4000 cps in a plastic bag for 20 min, followed by the addition of 5 mL of ethanol–water (60%:40%). The mixture was kneaded and passed through the 20-mesh sieve, followed by drying in an oven at 45C for 90 min. After drying, magnesium stearate (0.9%) was added. The resulting mixture was weighed and compressed (1 ton) by a hydraulic tablet press (DTP 25 v2 Desktop Tablet Press) with a round-shaped punch (diameter: 7.0 mm). All formulation batches were investigated to guarantee their quality attributes: friability test, disintegration time, uniformity of weight (mass and drug), and dissolution.

The concentration ranges for each factor were defined using preliminary formulation trials performed to establish practical and mechanistically relevant boundaries. These initial tests ensured (i) adequate tablet compressibility and mechanical integrity; (ii) formation of a stable gel layer suitable for extended release; and (iii) modulation of dissolution profiles across a range sufficient to support mathematical optimization against the Aricept^®^ reference product. Lactose monohydrate was used as a diluent to q.s. 750 mg to maintain a constant final tablet weight across all formulations (more details can be found in the Supplementary Materials).

### 4.4. In Vitro Dissolution

In vitro dissolution testing was conducted using the paddle method (USP Apparatus II) in 0.05 M phosphate buffer (pH 6.8) maintained at 37 °C. Two paddle rotation speeds, 50 and 100 rpm, were initially evaluated during method development. Samples (2 mL) were withdrawn at predetermined time points (0.5, 1, 2.5, 5, and 13 h), filtered through 0.45 µm polyethylene syringe filters, and analyzed via HPLC.

For the purposes of virtual bioequivalence (VBE) simulation and comparison with the reference product, a paddle speed of 100 rpm was selected. This condition provided greater discriminatory power in differentiating formulation performance compared to 50 rpm, enabling sensitivity to polymer-level variations within the optimized formulation space (unpublished internal development data). Therefore, all dissolution profiles used as inputs for the VBE assessment were obtained under 100 rpm conditions.

### 4.5. Dissolution Profile Similarity

Dissolution profiles were compared using the similarity factor (*f*2). The similarity factor is a function of the reciprocal of the mean square-root transform of the sum of square distances at all points and is a measure of the similarity in the percent rate of drug release between two dissolution profiles. The value of *f*2 ranges between 0 and 100, with a higher *f*2 value indicating more similarity between the two profiles. An *f*2 of more than 50 indicates that the dissolution profiles are similar to the marked reference formulation, with a mean % difference between dissolution profiles at exactly 10%. The similarity factor was calculated by the bootstrap method (Equation (1)) and is calculated against the reference formulation.

(2) $$f2=50\times log\{[1+\frac{1}{n}\sum_{n=1}^{n}{\left(R-T\right)}^{2\;}{]}^{-0.5\;\;\;}\times 100\}$$

### 4.6. Hydration and Erosion

Tablets were weighed (initial weight) and placed in 900 mL of 0.05 M phosphate buffer pH 6.8 and stirred during the dissolution test (paddle agitation speed 50 or 100 rpm); then, they were withdrawn from dissolution vessels at 1, 2.5, 5, and 13 h, weighted (wet weight), dried at 110 °C for 12 h, and reweighed (dry weight). The experiments were performed in triplicate.

(3) $$Hydration\left(\%\right)=\frac{wet\;weight-dray\;weigth}{dry\;weight\times 100}$$

(4) $$Erosion\left(\%\right)=\frac{inital\;weight-dray\;weigth}{initial\;weight\times 100}$$

### 4.7. Determination of Gel Strength upon Tablet Hydration

One planar base of the tablet was deep-coated with impermeable Eudragit^®^ RS (Haihang, Dezhou, China) dissolved in isopropanol and subsequently glued with the covered side of the tablet to the bottom of a small Petri-dish (diameter 70 mm). These samples were prepared as described in the item 0. The gel strength of swollen tablets at predetermined time intervals was measured using a texture analyzer (TA.XTplus, Stable Micro Systems Ltd., Surrey, UK) equipped with a 10 mm in diameter flat-tipped, round steel probe. The test conditions were that the pre-test speed was 0.2 mm/s, the trigger force was 0.1 g, and the test speed was 0.1 mm/s. Gel strength was calculated as the ratio between the penetrating force and the displacement of the probe inside the gel according to the following equation:

(5) $$G\;=\;\frac{Fx}{x}\frac{1}{rp}\times 0.0098$$

where G represents gel strength (MPa), F represents force (g) registered at probe penetration, x represents penetration depth (mm), and *rp* represents radius of the probe (7.0 mm). The gel strength at the gel–solution interface was considered as the first point after the probe was in full contact with the gel (trigger force reached) and the initial noise disappeared. We compared four formulations: Aricept XR (commercial brand), F_opt_ (our optimized formulation), F_slow_ slow release rate (5% of NaCMC, 40% of HPMC 100, and 40% of HPMC 4000), and F_fast_ fast release rate (5% of NaCMC, 5% of HPMC 100, and 5% of HPMC 4000).

### 4.8. PBBM Development

The model incorporated physicochemical parameters including molecular weight (M_W_), pKa, solubility, metabolism, octanol–water partition coefficient (logP_o:w), fraction unbound in plasma (f_u_), and blood-to-plasma ratio (B:P). Elimination kinetics were characterized using enzyme kinetics parameters (V_max_, K_m_), considering donepezil metabolism by CYP2D6 and CYP3A4, and the absorption model predicted by the ACAT model. All parameters, sourced from the existing literature, are detailed in Table 3. The baseline model was validated against observed pharmacokinetic profiles from nine oral immediate-release (IR) administration studies. The PBBM and Virtual Bioequivalence (VBE) evaluations were conducted using GastroPlus^TM^ version 9.9 (Simulations Plus, Inc., Research Triangle Park, NC, USA).

The model validation involved simulating multiple clinical studies with oral administration, comparing simulation outputs to mean pharmacokinetic profiles from published clinical data. Virtual populations were generated to match the demographic characteristics—ethnicity, gender, age, and body weight—of subjects in the referenced in vivo clinical trials. Additionally, the study design accounted for dosage forms and fluid intake volumes during administration.

For subsequent simulations, a controlled-release (CR) integral tablet dosage form was selected to represent the extended-release formulation. Dissolution profiles were input into the model as a Weibull function via GastroPlus’s (.crd) file to facilitate *in vivo* prediction.

#### 4.8.1. Criteria for PBBM Validation

To assess the model’s prediction accuracy for pharmacokinetic (PK) parameters, we utilized the Average Fold Error (AFE). It is defined by the equation:

(6) $$AFE\;=10^{\frac{1}{n}\sum \log\frac{{Predicted}_{i}}{{Observed}_{i}}}$$

The AFE is an indicator of the prediction bias. A method that predicted all observed values with no bias would have a value of 1; under-predictions are shown by an AFE below 1 and over-predictions by AFE values above 1. A prediction may be considered satisfactory if the AFE is between [0.8–1.25] [21]. Where *n* is number of studies.

#### 4.8.2. Parameter Sensitivity Analysis

We undertook a Parameter Sensitivity Analysis (PSA) to identify key parameters impacting the absorption rate and to gauge their influence on *in vivo* performance indicators, such as C_max_, T_max_, and AUC. The analysis involved individual parameters, considering both the formulation performance and the physiological.

Lag time (h)
Fmax (%)
Weibull shape parameters (Weibshape 1 and 2)
Weibull scale parameters (Weibscale 1 and 2)
Peff- effective permeability
Vmax- maximal rate of metabolism
Stomach transit time (SST).

#### 4.8.3. Virtual Bioequivalence

The VBE trials were structured as two-sequence, two-treatment, two-period crossover studies. The incorporation of within-subject variability (WSV) was enabled through a mechanistic method, termed “Simulate Physiologic Intrasubject Variability.” This approach implies that pharmacokinetic parameters were influenced by variations in individual physiology between occasions. The virtual population was built, and its variability was adjusted according to the methodology proposed by Kollipara and colleagues [10,35,42]. In summary, the workflow was as follows: First, we verified that the PBPK model could accurately replicate the pharmacokinetics observed in various clinical studies using the immediate-release (IR) formulation. Next, we identified key physiological parameters contributing to intra- and inter-subject variability and integrated these into the model. Third, we incorporated the formulation release profile into the model and compared the results with observed data. Once the PBBM successfully reproduced BE studies documented in the literature, we applied it to assess the likelihood of success for prototype formulations. A total of 15 trials for each formulation were tested. Success was categorized as follows: low risk (≥13 out of 15 trials meeting BE criteria), moderate risk (9–12 trials of 15), and high risk of failure (<9 trials meeting BE criteria).

## 5. Conclusions

This study successfully demonstrates that integrating Design of Experiments (DoE) with Physiologically Based Biopharmaceutics Modeling (PBBM) offers a robust and efficient pathway for developing extended-release formulations of donepezil. Using a systematic Box–Behnken design, we identified an optimized formulation that closely reproduces the dissolution profile of the reference product, Aricept^®^. In parallel, the PBBM framework provided accurate *in silico* predictions of in vivo pharmacokinetics and virtual bioequivalence (VBE), reinforcing the potential of model-informed tools to reduce reliance on extensive clinical trials.

However, in the specific case of donepezil extended-release (XR), a complete waiver of in vivo bioequivalence (BE) studies based solely on VBE remains unlikely under current regulatory expectations. FDA product-specific guidance continues to recommend multiple in vivo BE studies for this formulation type, reflecting the inherent complexity of extended-release technologies and their susceptibility to variable release kinetics, food effects, and dose-dumping risks. Although VBE and PBBM offer valuable supportive evidence, regulators generally require confirmatory in vivo data unless a robust, validated IVIVC and comprehensive in vitro characterization can be demonstrated. Although the developed model has significant credibility, the model could not be validated against human data due to the absence of a clinical study. Performing an actual human study is not within the scope of this paper, but we plan to conduct a human study in the future and validate the model against the clinical data. We plan to publish this work in our future manuscripts.

Despite these constraints, the combined DoE–PBBM strategy presented here clearly accelerates formulation optimization, reduces development costs, and enhances mechanistic understanding, ultimately facilitating regulatory compliance. Overall, our findings support broader implementation of model-informed drug development approaches across the pharmaceutical industry, particularly for generic and modified-release products where efficiency, predictability, and scientific justification are critical.

## Figures and Tables

**Table 1:** Summary of factor effects and significances (*p*) on the dissolution rate of donepezil in the development of extended-release tablets.

Factor		Model Coefficients		
	Y1 (1 h)	Y2 (2.5 h)	Y3 (5 h)	Y4 (13 h)
Model intercept	43 ***	92.0 ***	91.8 ***	92.0 ***
x1	−0.67 ***	−0.73 **	−0.72 **	.
x2	−0.96 ***	−1.37 ***	−1.36 **	.
x3	+1.9 **	+0.8 **	+0.02 **	.
x1.x2	.	.	.	−0.034 ***
x1^2^	+0.007 *	.	.	−0.01 *
x2^2^	+0.01 **	+0.02 **	+0.02 **	.
x3^2^	−0.19 **	.	.	+0.02 **

Note: HPMC 100 (x1), HPMC 4000 (x2), CMC (x3), linear interaction (x1, x2), quadratic interaction (x1^2^, x2^2^, and x3^2^). Signif. codes: <0001 ‘***’, 0.001 ‘**’, 0.01 ‘*’, ≥0.05 ‘.’. + and − to indicate the sign of the tendency effect (positive or negative).

**Table 2:** Summary of the adjusted parameter variability used in the model to match the observed in vivo between-subject and intra-subject variability. The table lists each parameter, its baseline value, the type of variability (BSV or WSV), and the final percentage range applied in the simulation.

Parameters	Default Variability	Inter-Subject Variability	Intra-Subject Variability
CYP34A Vmax	10	15	15
CYP2D6 Vmax	10	15	15
Stomach transit time	64	35	35
Peff	64	30	30
Weibscale 1 and 2	10	5 *	5 *
Weibshape 1 and 2	10	5 *	5 *
Fmax	10	3.2 *	3.2 *

* *in vitro* dissolution variability; the other physiological parameters were kept as software defaults.

**Table 3:** Donepezil PBPK model parameters used in the development of extended-release tablets.

Parameter	Values	Reference
Molecular weight (g/mol)	379.5	DrugBank (https://go.drugbank.com/drugs/DB00843 (accessed on 5 August 2024))
logPo:w	4.2	
Blood–Plasma ratio	0.78	
Fraction unbound (%)	4	
Drug solubility at pH 2 (mg/mL)	31	In house
Drug solubility at pH 4.5 (mg/mL)	47	
Drug solubility at pH 6.8 (mg/mL)	52	
pKa-base and (SolFactor)	8.12 (616)	Fitted using drug solubility at pH 2.0, 4.5, and 6.8
CYP2D6-Vmax (ng/min/mg protein)	125	(Lu et al., 2015) [40] SF Fitted using Tiseo et al [41] 4.8 mg solution oral dose.
CYP2D6 Km (µmol/L)	47	
CYP3A4-Vmax (ng/min/mg protein)	89 (SF:5.0)	
CYP3A4 Km (µmol/L)	44 (SF:5.0)	
Distribution method	Full body PBPK, Lukacova method of Kp prediction for all tissues	GastroPlus default method
Peff 10 × 10^−4^ cm/s	3.1	Predicted by ADMET predictor 11.0
Dissolution model	XR direct *in vitro* input; IR Johnson model	-

## Data Availability

The original contributions presented in this study are included in the article/supplementary material. Further inquiries can be directed to the corresponding author.

## Supplementary Materials

The following supporting information can be downloaded at https://www.mdpi.com/article/10.3390/ph18121894/s1: Figure S1: Simulated *in vivo* dissolution and absorption profiles for both the XR and IR formulations. Figure S2: Parameter Sensitivity Analysis for the development of donepezil extended-release tablets. Figure S3: Sampling size calculation. Figure S4: Virtual bioequivalence between formulation F7, F8, F9, F11 and F12 for extended release of donepezil and market product Aricept^®^. Figure S5: Comparison between the simulated pharmacokinetic (PK) profile of the Eisai formulation using its in vitro dissolution data, the published Eisai PK profile, and the simulated PK profile of the optimized XR formulation. Table S1: Factors and their levels in the DoE to study the impact of excipients on the dissolution and virtual bioequivalence of donepezil extended-release tablets.
