# Supplementary material for: DoE- and PBBM-Driven Formulation Development of an Extended-Release Donepezil Tablet
Source: Pharmaceuticals (Basel). 2025 Dec 16;18(12):1894. doi: 10.3390/ph18121894 (PMC12735571; doi:10.3390/ph18121894)
Supplement: Supplementary file 1 [file pharmaceuticals-18-01894-s001.zip › pharmaceuticals-3967943-supplementary.pdf]

Article

DoE- and PBBM-Driven Formulation Development of an Extended-Release Donepezil Tablet

Frederico Severino Martins [1*], Leonardo Luiz Borges [2,3], Sivacharan Kollipara [4] , Praveen Sivadasu [4] and Renê Oliveira do Couto [4]

1-Institute of Technology and Research (ITP), Av. Murilo Dantas, 300, 49010-390 Aracaju, Sergipe, Brazil
2-School of Medical and Life Sciences, Pontifical Catholic University of Goiás, Goiânia 74605-010, GO, Brazil
3-Theoretical and Structural Chemistry Group of Anápolis, State University of Goiás, Anápolis 75132-903, GO, Brazil
4-Department of Pharmacy, Koneru Lakshmaiah Education Foundation, Green Fields, Vaddeswaram, Andhra Pradesh, 522302, India
5-Midwest Campus, Universidade Federal de São João del-Rei (UFSJ), Divinopolis 35501-296, MG, Brazil
* Correspondence: Fredseverinomartins@gmail.com

Abstract

This study explores the integration of Design of Experiments (DoE) with Physiologically Based Biopharmaceutics Modeling (PBBM) to streamline the development of extended-release (XR) formulations. Using donepezil (DZP) as a model drug, we developed an optimized XR formulation exhibiting a dissolution profile comparable to the reference product, Aricept®. A Box-Behnken experimental design was applied to systematically evaluate how formulation variables—HPMC100, HPMC4000, and NaCMC—affect drug release kinetics, tablet hydration, and erosion. This strategy enabled the identification of optimal excipient concentrations with minimal experimental effort. The *in vitro* dissolution data were then integrated into a PBBM framework to simulate drug release and pharmacokinetics, enabling virtual bioequivalence (VBE) assessments.The combined approach provided robust predictive insights into formulation performance, substantially reducing reliance on resource-intensive *in vivo* studies. Beyond its successful application with DZP, this integrated methodology offers a scalable and generalizable strategy for efficiently developing bioequivalent XR formulations for various clinically relevant drugs. Our findings highlight the importance of leveraging advanced statistical methods and in silico modeling to overcome contemporary pharmaceutical development challenges, paving the way for innovative, cost-effective solutions that significantly accelerate time-to-market.

Keywords: Design of Experiments; Physiologically Based Biopharmaceutics Modeling; extended-release formulations; Box-Behnken design; virtual bioequivalence

Table S1: Factors and their levels in the DoE to study the impact of excipients on the dissolution and virtual bioequivalence of donepezil extended-release tablets

**Factor**	**Levels**		
	-1	0	1
HPMC100 (%)	0	20	40
HPMC4000 (%)	0	20	40
NaCMC (%)	0	5	10

Figure S1: simulated *in vivo* dissolution and absorption profiles for both the XR and IR formulations.

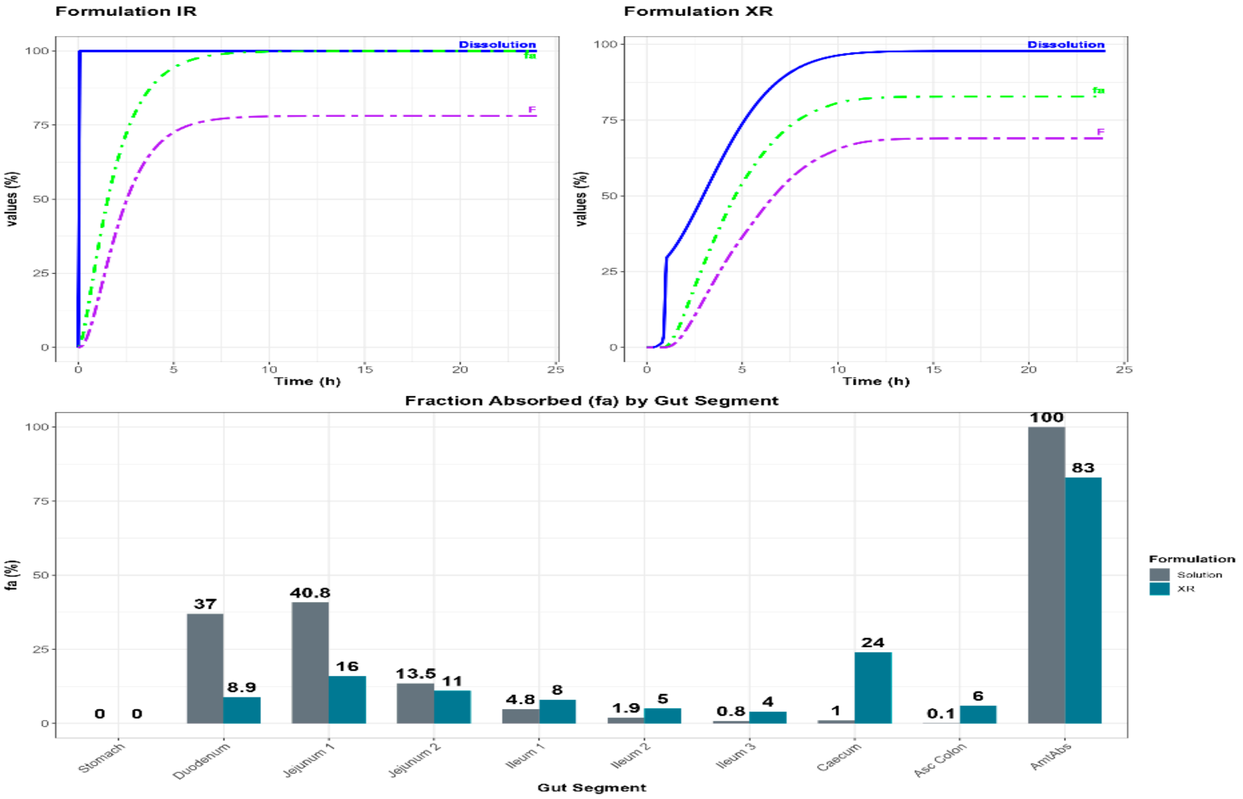

Figure S2 : Parameter Sensitivity Analysis for the development of donepezil extended-release tablets

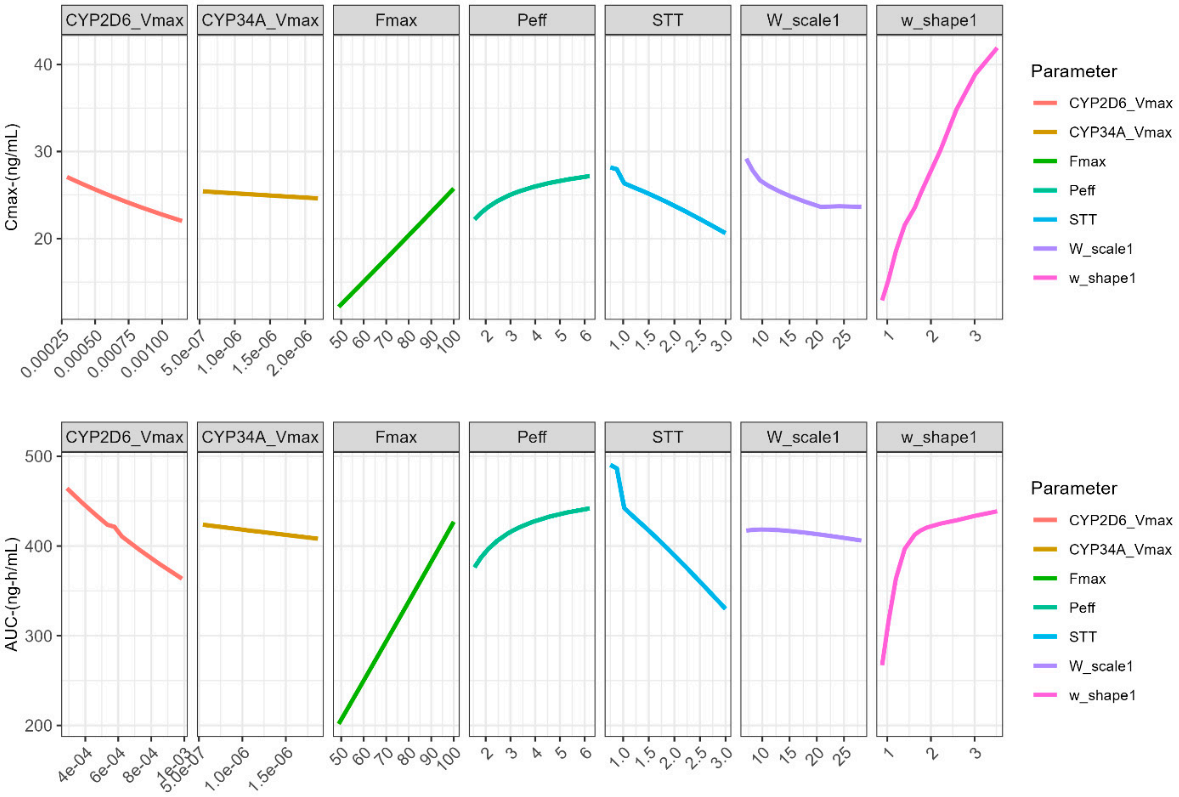
CYP2D6_Vmax
CYP34A_Vmax
Fmax
Peff
STT
W_scale1
w_shape1
Cmax-(ng/mL)
40
30
20
0.00025
0.00050
0.00075
0.00100
5.0e-07
1.0e-06
1.5e-06
2.0e-06
50 60 70 80 90 100
2 3 4 5 6
1.0 1.5 2.0 2.5 3.0
10 15 20 25
1 2 3
Parameter
CYP2D6_Vmax
CYP34A_Vmax
Fmax
Peff
STT
W_scale1
w_shape1
AUC-(ng-h/mL)
500
400
300
200
4e-04
6e-04
8e-04
1e-03
5.0e-07
1.0e-06
1.5e-06
50 60 70 80 90 100
2 3 4 5 6
1.0 1.5 2.0 2.5 3.0
10 15 20 25
1 2 3
Parameter
CYP2D6_Vmax
CYP34A_Vmax
Fmax
Peff
STT
W_scale1
w_shape1

Figure S3: Sampling size calculation

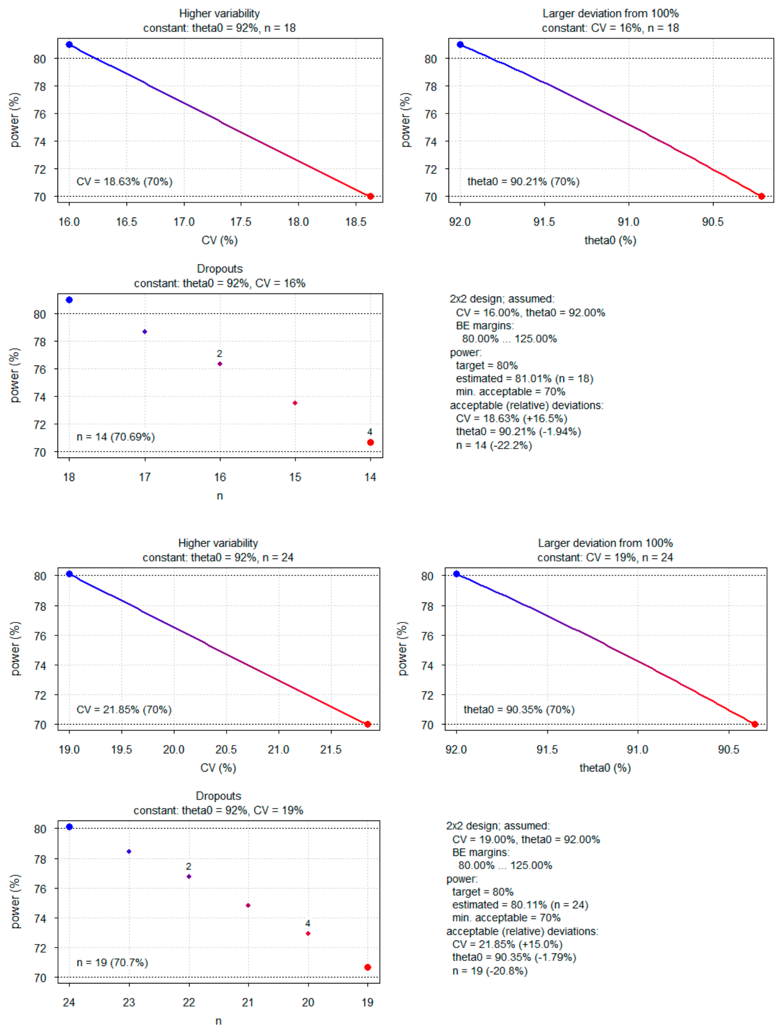

Figure S4 : Virtual bioequivalence between formulation F7 ,F8, F9, F11 and F12 for extended release of donepezil and market product Aricept®.

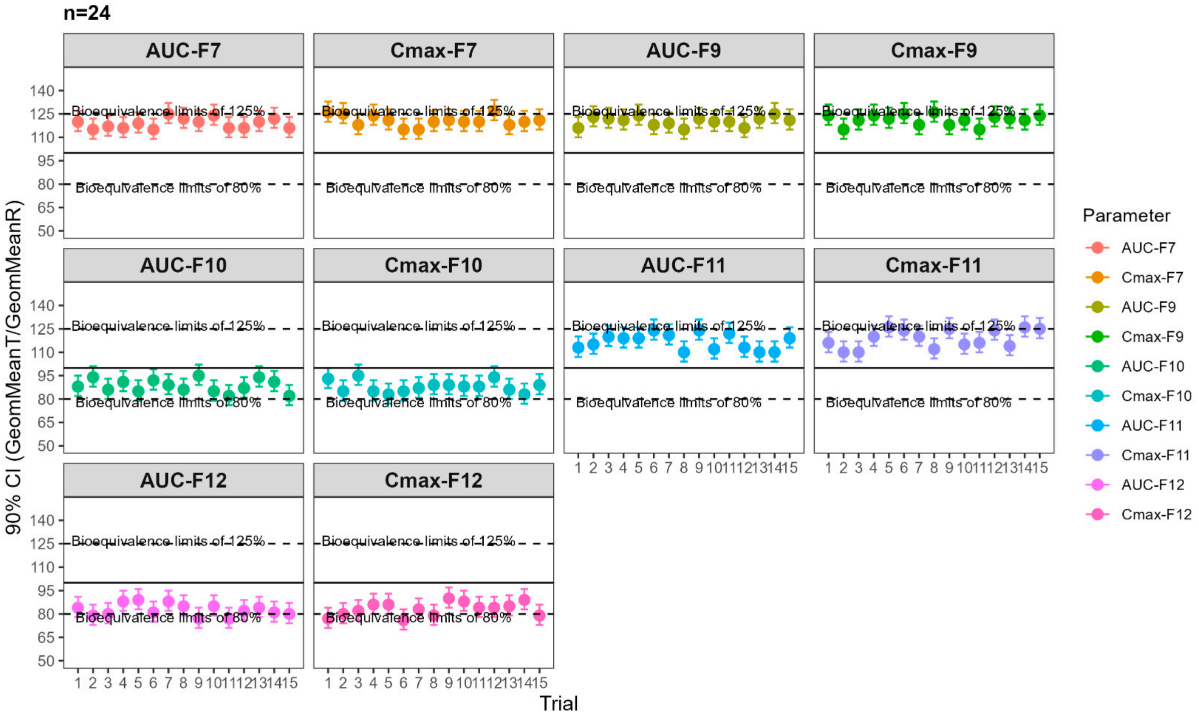

Figure S5: Comparison between the simulated pharmacokinetic (PK) profile of the Eisai formulation using its in vitro dissolution data, the published Eisai PK profile, and the simulated PK profile of the optimized XR formulation.

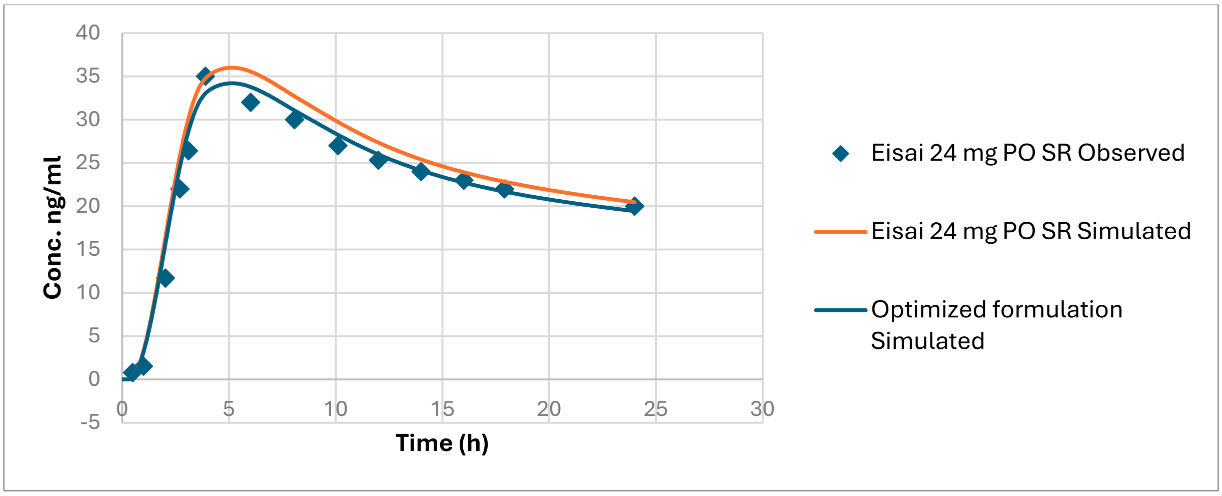

Prediction error between observed PK and Simulated

AUCped	AUCobs	MFEAUC	Cmaxped	Cmaxobs	MFECmax	Study	Fast/fed
758	612	0.807388	8.35	7.42	0.888623	Tiose 4.6 mg PO	Fasted
160	364	2.275	4.5	4.1	0.911111	Roger 1998 2 mg PO	Fasted
607	594	0.978583	9.8	8.2	0.836735	Roger 1998 4 mg PO	Fasted
695	661	0.951079	14	12	0.857143	Roger 1998 6 mg PO	Fasted
1286	1023	0.79549	24	25	1.041667	Choi 2015 10 mg PO Fr	Fasted
1286	1023	0.79549	24	25	1.041667	Choi 2015 10 mg PO Ft	Fasted
1368	1081	0.790205	25	23	0.92	Eisai 10 mg PO IR	Fasted
1130	1020	0.902655	21	21	1	Eisai 14 mg PO SR	Fasted
1981	1832	0.924785	36	32	0.888889	Eisai 24 mg PO SR	Fasted

Justification for Factor Selection and Level Ranges

The formulation variables included in the Box–Behnken Design (BBD)—HPMC 100 cps (X_1), HPMC 4000 cps (X_2), and NaCMC (X_3)—were selected based on their established roles as key release-modifying excipients in hydrophilic matrix tablets. HPMC polymers with distinct viscosities contribute differently to gel layer formation, water uptake, and diffusional resistance, whereas NaCMC affects matrix hydration dynamics and early-phase dissolution through ionic and swelling interactions. These mechanistic effects are consistent with prior literature on controlled-release matrices and were also supported by our previous work integrating machine learning and physiologically based biopharmaceutic modeling in sustained-release formulations (Severino Martins et al., 2023).

The concentration ranges for each factor were defined using preliminary formulation trials performed to establish practical and mechanistically relevant boundaries. These initial tests ensured (i) adequate tablet compressibility and mechanical integrity; (ii) formation of a stable gel layer suitable for extended release; and (iii) modulation of dissolution profiles across a range sufficient to support mathematical optimization against the Aricept® reference product. Lactose monohydrate was used as a diluent to q.s. 750 mg to maintain a constant final tablet weight across all formulations.

A 3-factor, 3-level Box–Behnken design was selected due to its efficiency in modeling nonlinear and interaction effects with fewer experimental runs compared to full factorial or central composite designs. The BBD avoids extreme corner-point combinations that may lead to non-formable or structurally unstable matrices, which is particularly advantageous when working with high polymer loads in extended-release hydrophilic systems.

Seventeen experimental runs were conducted, including five replicated center points. These center replicates served two purposes:

1. Statistical relevance – providing pure error estimation, enabling assessment of model fit, reproducibility, and curvature.
2. Formulation relevance – the center-point composition corresponded to a balanced polymer ratio that produced dissolution profiles closely aligned with the Aricept® reference product, reinforcing its suitability as an optimization anchor.

Dataset used in the modelling and simulation

Model application	Study description	Reference
Development	Formulation: Solution<br>Route: Oral<br>Population: HV<br>Fasted	Tiose et al 1998
Validation	Formulation: IR 10 , 14 mg, XR 24 mg<br>Route: Oral<br>Population: HV<br>Fasted	Esai monography
Validation	Formulation: IR 2 , 4mg, 6 mg<br>Route: Oral<br>Population: HV<br>Fasted	Roger 1998
Validation	Formulation: 2 IR formulations<br>Route: Oral<br>Population: HV<br>Fasted	Choi et al 2015

Modelling workflow

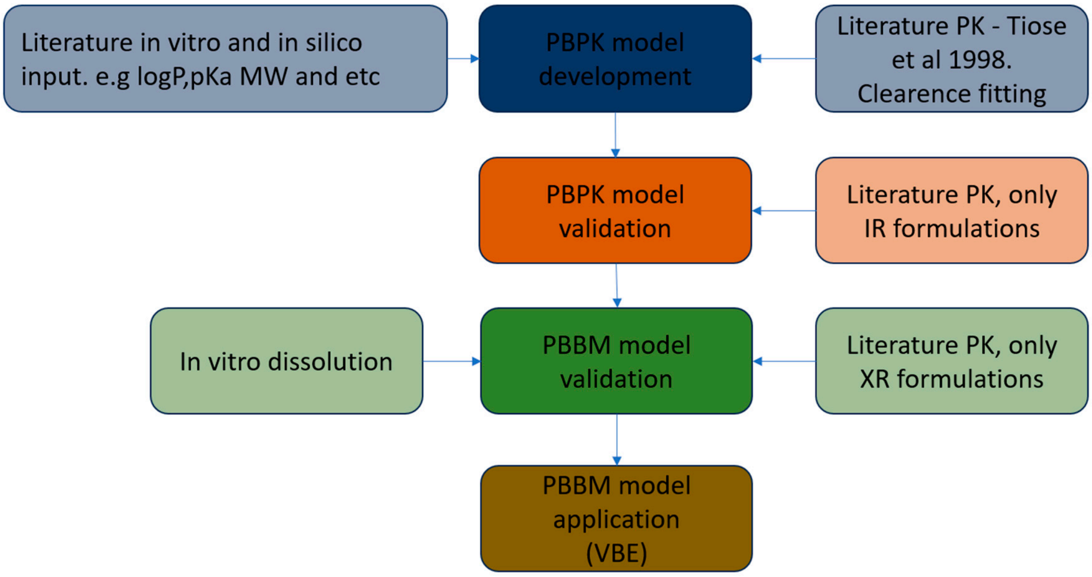

Literature in vitro and in silico input. e.g logP,pKa MW and etc
PBPK model development
Literature PK - Tiose et al 1998. Clearence fitting
PBPK model validation
Literature PK, only IR formulations
In vitro dissolution
PBBM model validation
Literature PK, only XR formulations
PBBM model application (VBE)
